# A Comparison of Cognitive Performance, Depressive Symptoms, and Incidence of Falls in Brazilian Older Women with and without a Confirmed History of COVID-19

**DOI:** 10.3390/ijerph20186760

**Published:** 2023-09-14

**Authors:** Marcelo de Maio Nascimento, Naiara de Souza Barros, Josiane Maria Rodrigues Coelho, Ana Beatriz dos Santos Silva, Adriane de Souza Ribeiro, Shákia Thâmara Guedes da Rocha Santos, Astrid Bibiana Rodríguez, Andreas Ihle

**Affiliations:** 1 Department of Physical Education, Federal University of Vale do São Francisco, Petrolina 56304-917, Brazil; 2 Department of Medicine, Federal University of Vale do São Francisco, Petrolina 56304-917, Brazil; naiara.souzab@discente.univasf.edu.br; 3 Department of Psychology, Federal University of Vale do São Francisco, Petrolina 56304-917, Brazil; josiane.coelho@discente.univasf.edu.br (J.M.R.C.); adriane.ribeiro@discente.univasf.edu.br (A.d.S.R.); shakia.guedes@discente.univasf.edu.br (S.T.G.d.R.S.); 4 Department of Pharmacy, Federal University of Vale do São Francisco, Petrolina 56304-917, Brazil; ana.beatrizsantos@discente.univasf.edu.br; 5 Department of Physical Education, National Pedagogical University, Bogotá 110221, Colombia; abrodriguez@pedagogica.edu.co; 6 Department of Psychology, University of Geneva, 1205 Geneva, Switzerland; andreas.ihle@unige.ch; 7 Center for the Interdisciplinary Study of Gerontology and Vulnerability, University of Geneva, 1205 Geneva, Switzerland; 8 Swiss Centre of Expertise in Life Course Research LIVES, 1205 Geneva, Switzerland

**Keywords:** SARS-CoV-2, COVID-19 pandemic, women, aging, cognitive functions, depression, vulnerability

## Abstract

The study aimed to compare cognitive performance, depressive symptoms, and the incidence of falls in Brazilian older women with and without a confirmed history of COVID-19. This cross-sectional study included 188 women (60–89 years), divided into two groups: one with a history of COVID-19 (n = 139), and one without any history of COVID-19 (n = 49). The instruments used were the Cognitive Telephone Screening Instrument (COGTEL) test battery, the Trail Making Test (TMT), the Geriatric Depression Scale (GDS-15), and the self-reported history of falls since the beginning of mandatory confinement. The higher the age, the higher the incidence of falls. The highest prevalence of falls (57.1%) occurred in the COVID-19 group (*p* = 0.001), the members of which also indicated a better cognitive performance in the COGTEL test (*p* = 0.017), TMT-B (*p* = 0.004), and ∆TMT (B-A) (*p* = 0.004). In turn, the depressive symptoms were more severe in the COVID-19 group (*p* < 0.001). We observed that COVID-19 infection without hospitalization did not affect the cognitive performance of older adult women. Future studies should be carried out to monitor the mental health of older adult Brazilian women. Moreover, regardless of their history of COVID-19, older adults should participate in a physical training program focused on preventing falls.

## 1. Introduction

In Brazil, the first confirmed case of COVID-19 occurred on 26 February 2020 [1]. Studies show that the incidence of the virus was high among different age groups; however, the fatality and mortality rate was higher in the older adult population [2,3]. In Brazil, 69.3% of deaths occurred in individuals over 60 years old, which was attributed to the presence of associated morbidities [4]. The relationship between the increased risk of death from the virus and age was enhanced via immunosenescence, which made this population more vulnerable to infectious diseases [5].

Studies have shown that individuals diagnosed with acute and subacute conditions of COVID-19, after healing, have a series of neurological sequelae, such as myalgias, headache, dizziness, hypogeusia, hyposmia, polyneuropathy, myositis, cerebrovascular diseases, encephalitis, and encephalopathy [6,7,8]. Viral infections can reach the central nervous system, causing neuropsychiatric syndromes affecting the cognitive, behavioral, and perceptual functions [9]. However, there are still gaps regarding the association between COVID-19 infection and cognitive deficits, mainly in relation to the intensity of cognitive alteration, according to the severity of the respiratory symptoms [10]. It is known that cognitive problems are more frequent in patients who have had a prolonged hospital stay, or even intubation [11]. However, in the case of those with mild symptoms, or who were not hospitalized, it is unclear whether they have cognitive deficits, when objectively measured [12].

Another health impact potentiated by SARS-CoV-2 was a series of neuropsychological changes. In general, widespread outbreaks of infectious diseases, such as COVID-19, were associated with a high degree of psychological distress, responsible for symptoms of mental illness [13]. Studies have reported mental problems, such as stress, anxiety, depression, mood swings, sleep quality, and fear [14,15,16]. Moreover, during, and shortly after, the pandemic period, there was a relative increase in psychotic disorders, due to worry [17]. Both for individuals who were hospitalized, especially those for a long time, and for those who performed self-isolation in their homes, the time spent alone proved to be inversely proportional to the consequences for mental health [6]. Moreover, after exposure to COVID-19, there was an increase in the prevalence of post-traumatic symptoms [18].

Until immunizations were developed, staying at home and adopting strict hygiene measures were the only alternatives capable of preventing the spread of the virus and saving lives [19,20]. Thus, if, on the one hand, preventive measures such as self-isolation, quarantine, shielding, and blockade contributed to saving lives, on the other hand, they impacted the physical and mental health of populations [21,22]. Moreover, it is known that these changes were even greater in older adult individuals [23,24]. This occurred because “staying at home” created strong barriers to maintaining social bonds, which aroused feelings of abandonment and loneliness, and negatively reflected on mental health, quality of life, and wellbeing [25]. In addition, the fact that they remained confined to their homes drastically reduced the physical activity level of older adults, making them even more sedentary [26,27]. Confinement caused a sudden decline in mobility. Thus, older adults became more fragile, and susceptible to several diseases [28].

According to the specialized literature, in general, older adults tend to present a progressive cognitive decline [29]. Thus, it is possible that both the virus infection and the long period of social isolation affected the cognitive functions of this subgroup, even those considered cognitively normal [30]. Thus, factors associated with sudden changes in lifestyle, such as a reduction in the level of physical activity, a lack of stimulating schedules and daily activities, and the breaking down of social networks, may have affected the cognitive performance of older adults [31]. Another issue that deserves further study is the verification of possible cognitive and mental impacts in older women after infection with COVID-19, as well as a comparison of this group with older women who have not been infected by the virus. Previous studies have highlighted that older women are more likely to develop depressive symptoms than younger women, as well as men in the same age group [32,33]. Older women are at a greater risk of falling than young women and older men [34]. Moreover, there is a direct relationship between depression and an increased risk of falls [35]. When it comes to the older population, falls are a public health problem, responsible for fractures and hospitalization days, as well as the death of one in every three older adult fallers [36].

Even today, the overall impacts of COVID-19 on different populations are unknown. However, studies have highlighted that there are complaints about impaired cognitive performance among those who contract the virus at a level classified as an acute or subacute degree of infection [16,17]. On the other hand, when it comes to the older population who contracted coronavirus, but did not require hospitalization, and recovered from the disease through self-quarantine, there are gaps regarding the late evolution of problems in cognitive functions and mental changes [12]. Another question to investigate regarding the effects of COVID-19 on populations is the evidence that the virus not only showed genetic diversity, but also evolved rapidly in different regions of the world [37]. Therefore, its performance characteristics may vary according to the demographic and epidemiological profile of each location and population [38]. Thus, it is necessary to research and disseminate information that makes us more aware of the virus. This measure can strengthen shared knowledge, contributing to the creation of effective strategies, in case new pandemics arise. Moreover, gaining and sharing knowledge can prevent high mortality rates from recurring. This is fundamental, mainly for regions of greater social and economic vulnerability (i.e., developing countries), such as the Northeast Region of Brazil, the focus of this study [39,40]. The present study aimed to compare the cognitive performance, depressive symptoms, and incidence of falls in Brazilian older women with and without a confirmed history of COVID-19.

## 2. Materials and Methods

### 2.1. Study Design and Eligibility

This is a cross-sectional analytical observational study. The sample was selected for convenience because, due to the barriers imposed by the confinement of COVID-19, it was not possible to include a large number of participants in the study. Among the impediments, we highlight direct access to older adults, the dependence on potential participants answering phone calls, owning devices with cameras, knowing how to use these devices, remembering the days scheduled for the interviews, signing the terms of participation for the study, as well as sending this document to our team via email or WhatsApp. Finally, 188 women (60–89 years old) were included. All participants lived in the city of Petrolina (388,145 inhabitants), in the Northeast Region of Brazil. This city is located in an inland region, 713 km from the city of Recife (1,653,461 inhabitants), the capital of the state of Pernambuco [41]. Participants were recruited between July 2020 and February 2021, through phone calls. They were former students of the Open University of the Third Age at the Federal University of Vale do São Francisco. The inclusion criteria were as follows: aged between 60 and 90 years, living in the community, and with no limitation in understanding or following the evaluation protocol. The study participants were informed about the objectives and risks of the investigation. Prior to providing data or undergoing any assessments, those who agreed to participate signed a consent form in accordance with the Declaration of Helsinki. The participants of the present study received no financial compensation.

### 2.2. Data Collection

#### Demographics and Clinical Data

Through phone interviews or video calls, the participants answered a questionnaire on their sociodemographic data (i.e., age, years of education), number of different types of medication consumed daily, comorbidities, and history of falls. Two questions were applied to investigate falls. The first was: “During the confinement period, more precisely between July 2020 and February 2021, did you suffer any falls?”. The second question was: “Before the confinement period, more precisely, in a period of up to 12 months, did you suffer any falls?”. Due to the recall problems and uncertainty of most participants, it was not possible to determine the exact location of falls prior to the confinement period. Regarding the coronavirus, the question asked was whether the participant had contracted the coronavirus (yes/no). However, only cases confirmed through a laboratory test for SARS-CoV-2, such as real-time reverse transcriptase PCR (positive RT-PCR) were considered for the analysis. Therefore, if the answer was yes, the participant was asked about hospitalization, and how many days. Considering that none of the participants infected with COVID-19 needed to be hospitalized (the following table). The interviews were conducted by field staff members specially trained to follow COVID-19 health prevention procedures [42].

### 2.3. Instruments

#### 2.3.1. Cognitive Assessment

To measure cognitive functioning, we used two instruments: (1) the Cognitive Telephone Screening Instrument (COGTEL) test battery [43], and (2) the Trail Making Test (TMT) [44]:

The COGTEL has an excellent reliability and high validity, and the psychometric properties were verified in the Brazilian older adult population [43]. In the present study, the COGTEL was applied through a telephone call, during a previously scheduled session. The average test application time was approximately from 10 to 16 min. The assessment of cognitive functions takes place through six subtests: (1) prospective memory; (2) short-term verbal memory; (3) working memory; (4) inductive reasoning; (5) verbal fluency; and (6) long-term verbal memory. In the prospective memory subtest, participants were instructed to say, out loud, the year of their birth, when the interviewer spoke the following signal phrase during the assessment: “Please try to name as many professions as possible”. This phrase is part of the verbal fluency assessment subtest, receiving a dichotomous prospective memory score. Thus, the score used was as follows: 1 = approved or 0 = not approved. The short-term verbal memory subtest requires the immediate recall of eight word-pairs. Thus, the participants have to learn the word-pairs; for the evocation of the second word, there was a signal through the first word of the respective pair (0 to 8 points). In the long-term verbal memory subtest, there was delayed recall of these eight pairs of words (0 to 8 points). In the working memory subtest, participants were asked to listen to 12 progressively longer sequences of single-digit numbers. Afterwards, they were asked to immediately reproduce each of the sequences aloud; however, the digits should be reproduced in reverse order to the one presented initially (0 to 12 points). The verbal fluency assessment consisted of naming the maximum number of words possible in one minute, starting with the letter A (letter fluency), as well as the largest possible number of professions (category fluency), also in one minute (from 0 points to unlimited; derived from the sum of the words the participant produced during the two evaluations). Finally, in the inductive reasoning subtest, participants were asked to listen to eight sequences of five numbers (constructed following a specific mathematical rule). The task consisted of completing each of these sequences by adding a sixth number, through detecting the respective underlying rule on their own (from 0 to 8 points).

The calculation of the total test score is obtained via a weighted average [45]: COGTEL total score = 7.2 × prospective memory + 1.0 × short-term verbal memory + 0.9 × long-term verbal memory + 0.8 × working memory + 0.2 × verbal fluency + 1.7 × inductive reasoning. The deficit in cognitive performance was established via subtracting the general average of the total COGTEL (score = 14.41 points) minus 1 standard deviation, which determined a COGTEL cutoff point < 13.41 score. This calculation is used to identify individuals with a cognitive vulnerability or mild cognitive impairment [46].

The TMT test consists of two parts (A and B), one with numbers, and the other with numbers and letters [47]. In the TMT-A, participants were asked to draw a line with a pen as quickly as possible on the paper, connecting, in ascending order along the way, circled numbers from 1 to 25, posted at random. The task is considered as visual velocity scanning; therefore, it requires motor coordination and visuospatial processing. In TMT-B, the task consisted of connecting the same number of circles. However, the displayed sequence presented alternating numbers and letters (1, A, 2, B, 3, C...). This test assessed working memory, cognitive flexibility, and switching settings and tasks. Both tests were timed. Thus, the faster (measured in seconds) the test execution, the better the cognitive performance. We also calculated the difference in scores (∆TMT = TMT-B score − TMT-A score). Considering that Part B is more complex than Part A [48], a high ∆TMT score is indicative of a possible alteration in executive functions [49].

In the present study, the text of the TMT (two copies) was sent to the participants by post, accompanied by the terms of consent for participation in the study, which were signed before any study questionnaire was carried out. Considering the social distancing norms established during the coronavirus pandemic, the TMT was administered through a videoconference. Initially, the participants were asked to place the smartphone on the table, so that the interviewer could observe both the paper (test trails) and the pen during the test execution. Two attempts were made for Part A and another two for Part B. The first execution consisted of an abbreviated version of the TMT (Part A and B) to clarify possible doubts. In the second execution, the evaluators record the time taken to perform the test to assess cognitive performance. Prior to the adoption of the video-calling procedure for the TMT test, the executing team conducted a pilot with 28 older adults, to improve this test execution technique. The participants in the pilot test procedures were not part of the final study sample.

#### 2.3.2. Depression

The Geriatric Depression Scale (GDS-15), one of the most used self-reports of depression in the scientific field, was used to examine the somatic complaints of older adult individuals [50]. Its items are short, easy to understand for the older adult population, and suitable for all levels of education, in addition to including important age-related information. The GDS-15 scores were interpreted as follows: (1) score ≤ 5 = no depressive symptoms, (2) score from 6 to 10 = mild depression, and (3) score ≥ 11 = severe depression [51].

### 2.4. Statistics

The sample was divided into two groups: women diagnosed with coronavirus infection (n = 49), and those without COVID-19 infection to date, at the time of assessment (n = 139). The data distribution was tested using the Shapiro–Wilk test. The demographic data (numerical variables) were presented as mean and standard deviation. Categorical variables were presented by several cases and proportion (%), using the chi-square test for the comparison of the difference between the groups. Continuous variables with a normal distribution (COGTEL total score, TMT A, TMT B, TMT B-A, GDS) were presented as means and standard deviations, and analyzed using the unpaired Student’s *t*-test for independent samples. For quantitative variables without a normal-distribution COGTEL subscale (short-term memory, long-term memory, working memory, verbal fluency, reasoning), the median and interquartile range (IQ) were calculated, and subsequently analyzed via the non-parametric Z Kolmogorov–Smirnov test. In the case of the “prospective memory” subscale, as it is dichotomous (yes/no), the results were presented by the number of cases and proportion (%), while the difference between groups was calculated using the chi-square test.

## 3. Results

In the present study, the average age of the population was 70.84 ± 6.00 years. Of the 188 women who participated in the analyses, 26.1% belonged to the group diagnosed with COVID-19. A statistical difference in the evaluations of the main characteristics was found only for the COVID-19 group, which showed a prevalence of 20.4% for self-reported cancer disease (*p* = 0.008) (Table 1).

Table 2 presents, in a comparative way, the performance results of the study participants in the variables of interest in the study. As for the history of falls during the mandatory confinement for coronavirus, there was a rate of 37.2% for the entire sample. Therefore, members of the COVID-19 group showed a higher prevalence of falls (57.1%). (*p* = 0.001). Moreover, it was found that, in both groups, the higher the age, the higher the incidence of falls. Regarding the total score in the COGTEL test, the COVID-19 group showed a better cognitive performance (*t*(186) = 2.406; *p* = 0.017). The findings for the six subscales of the COGTEL test were as follows: prospective memory (χ^2^(1) = 5.746; *p* = 0.017), short-term memory (Z = 0.192; *p* = 0.139), long-term memory (Z = 0.067; *p* = 0.997), working memory (Z = 0.178; *p* = 0.199), verbal fluency (Z = 0.234; *p* = 0.037), and reasoning (Z = 0.275; *p* = 0.008). The comparative analysis showed that the prevalence of cognitive impairment in older adult women with COVID-19 was inferred (32.7%) from those without a history of COVID-19 (51.1%). The second cognitive test applied was the TMT. In Part A, there was no significant difference (*t*(186) = 0.855; *p* = 394). On the other hand, significantly better performance results were observed in the COVID-19 group in the evaluation of the TMT-B (*t*(186) = 2.967; *p* = 0.004) and ΔTMT (B-A) (*t*(186) = 3.011; *p* = 0.003). Regarding the self-reported depressive symptoms, the COVID-19 group showed a higher total score on the GDS-15 (*t*(186) = 3949; *p* < 0.001). Proportionally, mild depression symptoms were observed in the majority (75.6%) of the non-COVID-19 group, while the majority (60.9%) of older adult women with a history of coronavirus indicated severe depression.

## 4. Discussion

Our study aimed to compare cognitive performance, depressive symptoms, and the incidence of falls in Brazilian older women with and without a confirmed history of COVID-19. The findings showed a prevalence of falls of 57.1% for members of the COVID-19 group during the period of mandatory confinement. Moreover, it was found that the higher the age group, the higher the prevalence of falls. Regarding cognitive performance, except for the TMT Part A test (*p* > 0.050), comparatively, members of the COVID-19 group showed a better cognitive performance in all other assessments. Finally, we confirmed that confinement was a stressful period for older adult women, 100% of the members with COVID-19, and 93.3% of those without COVID-19, attested to experiencing depressive symptoms.

Regarding falls, our findings were in line with previous studies carried out during the confinement period. A meta-analysis study that included investigations published between December 2019 and July 2020 (n = 27) to verify a possible correlation between COVID-19 and diseases of the musculoskeletal system, in particular the occurrence of injuries related to falls [52], highlighted that, during the pandemic, low-energy injuries (i.e., falls, slips) were the most common cases of wounds among older adults. Our findings do not allow us to state whether the observed fall events were directly caused by the coronavirus. However, the literature highlighted atypical associations between falls and coronavirus, especially in the older adult population [52]. Thus, when factors such as muscle strength deficit due to physical inactivity caused by confinement are added to a sudden drop in blood pressure and fatigue, dizziness can be generated, which, in turn, can be added to drug interactions, and delusions [52]. All this can potentiate problems in postural control, which is essential to maintaining an upright posture [53,54]. In turn, small changes in the sensory system (i.e., vision, hearing, and the somatosensory system) can considerably affect the sensory regulation of static and dynamic balance [55], limiting the flow of information and responses at the afferent and efferent levels between the extremities of the body and the brain [56], increasing, consequently, the risk of falls [57].

The importance of studying falls during the pandemic stems from the fact that falls are more prevalent in women than in men [58]. Moreover, falls are considered a public health issue, due to the high risk of death after fractures [59]. In the present investigation, we did not obtain reports of fractures among those who fell. However, it is worth noting that, in the older adult population, there is an association between vitamin D deficiency and an increased risk of bone injuries after falls [60]. Due to the properties of vitamin D for the musculoskeletal system, especially during the pandemic, adequate levels of serum 25-hydroxyvitamin D (25(OH)D) are important in the aging process [61]. The 25(OH)D can contribute to the prevention of muscle fragility (preventing falls) and bone fractures [62]. Hypovitaminosis D has been associated with the vulnerability of older adults to respiratory infections [63], which, in the case of COVID-19, increases the risk of admission to an intensive care unit. However, it is noteworthy that the effectiveness of the supplementation of patients with COVID-19 or treatment with 25(OH)D is the subject of ongoing studies [64].

Regarding cognitive performance, studies carried out during the pandemic have found relationships between COVID-19 and poor cognitive performance in patients hospitalized due to severe cases of the disease [5,65]. However, it is important to highlight that cognitive impairment (CI) was found mainly in those older adults who already had CI. In a study carried out in Spain in March 2020 [66], among cases of patients with COVID-19 (n = 281; 81.4 years), 21.1% had dementia, and another 8.9% had mild CI. According to the authors, the patients with CI were older, lived in nursing homes, had a short lifespan from the onset of symptoms to death, and were rarely admitted to an intensive care unit. Moreover, in a study carried out in China with patients who survived COVID-19 (n = 1438; 66–74 years), after 12 months of cure, a CI incidence of 12.45% was verified [38].According to the authors, individuals who were previously classified as having severe symptoms of COVID-19 had lower scores for cognitive performance than those classified as non-severe, or without COVID-19. Additionally, a population-based study (n = 81,337) carried out in the UK showed significant cognitive deficits in hospitalized individuals who recovered from COVID-19, as well as in those not hospitalized [12].

Given the evidence, our results are not in line with previous studies, as members of the COVID-19 group indicated a better cognitive performance in objectively measured tests than individuals not infected with the virus. Members of the COVID-19 group indicated a better total COGTEL score, including three subcategories (i.e., prospective memory, verbal fluency, reasoning), as well as the TMT Part B and TMT B-A. A possible explanation for the non-compliance of our findings with the previous literature is that the sample consisted of cognitively healthy older adults. Moreover, the proportion between participants with COVID-19 (73.9%) and without COVID-19 (26.1%) was not homogeneous. Another point to highlight is that, comparatively, the prevalence of cognitive impairment detected via the COGTEL test was 18.4% higher among members of the group without COVID-19. All this suggests that the disparity in the number of individuals between the groups may be responsible for the better cognitive performance identified among older adult women with a history of COVID-19, residing in the city of Petrolina, Brazil. This finding also reinforces the need for the longitudinal monitoring of the performance of the cognitive functions of the members of both groups.

Although social isolation measures were necessary during the pandemic, to combat the spread of the virus, studies have highlighted the negative effects of this strategy around the world on the mental health of populations [67,68]. Among women, and especially older women, investigating the immediate and late psychological impact in response to the COVID-19 outbreak and quarantine is important because, comparatively, women are at a greater risk of psychological disorders than men [69,70]. Thus, our findings showed that 98.5% of the entire sample had mild-to-severe depressive symptoms between May 2021 and September 2021. Furthermore, our findings are in line with studies conducted in China [71], Japan [72], Ireland [73], Spain [67], the USA [74], Sweden [75], and even Brazil [27,76]. In a study carried out in Italy (n = 758; from 18 to 30 years) to investigate associations between age and sex in the evolution of mental health status after quarantine, as well as to investigate the baseline predictors of post-traumatic stress symptoms, severe CI was detected at follow-up (two months after the end of lockdown) [77]. The results suggested that psychological symptoms (i.e., depression, stress, anxiety, loneliness, moods) caused by social isolation impacted the cognitive functions of the assessed population. Moreover, Maggi et al. [77] pointed out that, due to the persistence of psychological symptoms two months after the end of lockdown, there would be a need to implement psychological support interventions, to avoid long-term mental health problems.

Our study has severe limitations. Firstly, the cross-sectional design does not allow for the generalization of the results to other populations, with and without a confirmed history of COVID-19, as well as to other women in the same age group living in the city of Petrolina, Brazil. However, our findings provided evidence for an initial understanding of the performance of cognitive functions, the occurrence of depressive symptoms, and the history of falls in a group of older adult women during the first 12 months of mandatory confinement due to the COVID-19 pandemic, living in a city far from a large urban center. Geographic information in the health area is fundamental to disease surveillance, management, analysis, and care [78]. Moreover, according to the Brazilian Society of Geriatrics and Gerontology [79], compared to other regions of the country, the older adult population from the North and Northeast regions are ageing with greater social and economic vulnerability. A second limitation of the study is the composition of the convenience sample. This is because, due to the pandemic’s safety regulations, our team faced barriers to contacting the local older adult population. Thirdly, the non-homogeneity between the number of participants in the groups with and without a history of COVID-19 may have generated bias. However, to verify the problems of homogeneity in the study, we performed both the Student’s *t* test and the Mann–Whitney test, noting that the results were identical. Finally, considering that the *t* test is robust to violations of the homogeneity of variances, we chose to advance the analyses with the parametric test. Furthermore, we emphasize that the non-homogeneity between the groups was a reflection of the security measures of the pandemic period. At the time, it was not possible to establish direct contact with the local older population, especially with those who had been infected with the coronavirus. Thus, many older adults did not agree to participate in the study, while others started to participate, but did not complete all the steps, being excluded from the sample. Fourthly, participation in the study required older adults to master the use of technological devices.

Fifthly, the lack of information about the history of depression and cognitive impairment among the participants is a serious limitation of the study. Furthermore, we did not rule out possible cases of reverse causality, when study members who already had depressive symptoms due to the stress caused by the pandemic did not report a mild COVID-19 infection, without the need for hospitalization. So, based on the findings, we suggest that the present study may stimulate future longitudinal investigations. Specifically, participants could be monitored to verify possible changes in their cognitive and mental health resulting from the COVID-19 confinement period. Sixthly, we must consider that, in the comparison between falls before confinement (up to 12 months) and during confinement, the members of the present study experienced a series of changes resulting from the natural process of physiological aging. Therefore, the time factor and its consequences, such as the increased risk of falls and changes in mental health, must be considered as a confounding factor that cannot be controlled in the analyses, and must be taken into account. Moreover, it is possible that participants missed minor falls without serious bone fractures. Thus, one should consider the possibility of the presence in the study of older adults who declared themselves non-fallers, but who had suffered a fall. Finally, we did not administer the PCR test to confirm the contagion of COVID-19, or its absence. Therefore, inclusion in one or another group was determined via self-report (yes/no). Thus, it is possible that some participants contracted the virus but remained asymptomatic. Moreover, in the case of the older adult population, which is a group with several chronic diseases [80], the association of SARS-CoV-2 with several diseases can mask sensory, cognitive, and psychological changes [81]. All this makes it difficult to identify the consequences left by the virus.

## 5. Conclusions

Our findings shed light on important information about cognitive performance, depressive symptoms, and the history of falls in Brazilian older women with and without a confirmed history of COVID-19. Unlike previous studies, we did not identify problems with cognitive performance in the COVID-19 group. Notably, regardless of whether older women living in the city of Petrolina, Brazil, had a confirmed or unconfirmed history of coronavirus, we identified, in the entire sample, a high prevalence of depressive symptoms and falls during the mandatory confinement. The information presented in this study can contribute to the understanding of the impacts of social isolation and the action of COVID-19 on the physical and mental health of older adult Brazilian women. Furthermore, the results serve as a warning to policymakers regarding the need to support the older adult population, cognitively and mentally, in the post-pandemic period. Among the options, we highlight holistic approaches, such as social, sport, or lifelong education groups. When it comes to mental health and falls, possible promotion strategies, especially for individuals who live alone or have accessibility problems, would be digital solutions, such as online therapies or the use of sensitive sensors capable of identifying falls, and notifying family members and support services. We also suggest carrying out comparative studies between investigations in developing countries and those in developed countries, so that the repercussions of the pandemic on vulnerable populations can be better understood and disseminated.

## Figures and Tables

**Table 1:** The main characteristics of the participants.

Variable	COVID-19 (n = 49)	No-COVID-19 (n = 139)	*p*-Value
Age (years)	70.89 ± 6.14	70.67 ± 5.64	0.827
60–69 (years)	18 (36.7)	61 (43.9)	
70–79 (years)	27 (55.1)	66 (47.90	
≥80 (years)	4 (8.2)	12 (8.6)	
Medication n (%)			0.138
1–4 types	48 (98.0)	130 (93.5)	
≥5 types	1 (2.0)	9 (6.5)	
Education n (%)			0.082
1–4 years	4 (8.2)	28 (20.1)	
5–8 years	12 (24.5)	30 (21.6)	
9–12 years	12 (24.5)	28 (20.1)	
≥13 years	21 (42.9)	53 (38.1)	
Physical exercise			
Yes (%)	45 (91.8)	117 (84.2)	0.135
Comorbidities n (%)			
Diabetes mellitus			
Yes (%)	9 (18.4)	34 (24.5)	0.253
Hypertension			
Yes (%)	30 (61.2)	85 (61.2)	0.566
Visual acuity			
Yes (%)	41 (83.7)	105 (75.5)	0.165
Hearing			
Yes (%)	11 (22.4)	32 (23.0)	0.553
Labyrinthitis			
Yes (%)	9 (18.4)	36 (25.9)	0.194
Osteoporosis			
Yes (%)	11 (22.4)	45 (32.4)	0.130
Rheumatism			
Yes (%)	10 (20.4)	31 (22.3)	0.478
Parkinson			
Yes (%)	-	2 (1.4)	0.546
Cancer			
Yes (%)	10 (20.4)	9 (6.5)	0.008 *

Data are expressed as mean (M) ± standard deviation (SD) or frequency (f); * *p* < 0.050.

**Table 2:** A comparison of the results of the COVID-19 and non-COVID-19 groups for assessments of falls, cognition, and depression.

Variable	COVID-19 (n = 49)	Non-COVID-19 (n = 139)	*p*-Value
Falls (during confinement)			0.001 *
Yes total n (%)	28 (57.1)	42 (30.2)	
60–69 years n (%)	8 (16.3)	11 (8.0)	
70–79 years n (%)	17 (34.7)	25 (18.0)	
80–89 years n (%)	3 (6.1)	6 (4.3)	
Falls (before confinement)			0.632
Yes total n (%)	9 (18.3)	25 (18.0)	
60–69 years n (%)	3 (6.1)	10 (7.2)	
70–79 years n (%)	5 (10.2)	13 (9.3)	
80–89 years n (%)	1 (2.0)	2 (1.4)	
Cognition			
COGTEL			
Prospective memory			0.009 *
** Approved n (%)	49 (100)	15 (10.8)	
^‡‡^ Short-term memory (n)	3 (1–5)	2 (0–6)	0.139
^‡‡^ Long-term memory (n)	3 (0–5)	3 (0–6)	0.997
^‡‡^ Working memory (n)	4 (0–7)	3 (0–8)	0.199
^‡‡^ Verbal fluency (n)	22 (4–50)	17 (1–39)	0.037 ^‡^
^‡‡^ Reasoning (n)	2 (0–8)	1 (0–6)	0.008 ^‡^
COGTEL total score (n)	16.37 ± 6.04	13.72 ± 6.82	0.017 ^†^
Cognitive impairment n (%)	16 (32.7)	71 (51.1)	
Normal cognition n (%)	33 (67.3)	68 (48.9)	
TMT-A (s)	63.42 ± 22.82	69.76 ± 39.90	0.394 ^†^
TMT-B (s)	147.42 ± 48.80	213.04 ± 122.85	0.004 ^†^
ΔTMT (B-A) (s)	84.00 ± 43.26	143.28 ± 109.37	0.003 ^†^
Depression			
GDS-15 (n)	10.70 ± 1.89	8.84 ± 1.80	<0.001 ^†^
score ≤ 5 (%)	-	9 (6.7)	
score 6 to 10 (%)	19 (39.1)	105 (75.6)	
score ≥ 11 (%)	30 (60.9)	25 (17.8)	

Data are expressed as mean (M) ± standard deviation (SD); TMT = Trail Making Test; GDS-15 = Geriatric Depression Scale; * = chi-square test; ^†^ = Student’s *t*-test for two independent samples; ^‡^ = Z Kolmogorov–Smirnov test; COGTEL = ** dichotomous score (yes/no), ^‡‡^ median and range.

## Data Availability

The data presented in this study are available upon request from the corresponding author.
